# Common Topological Features in Band Structure of RNiSb and RSb Compounds for R = Tb, Dy, Ho

**DOI:** 10.3390/ma16010242

**Published:** 2022-12-27

**Authors:** Semyon T. Baidak, Alexey V. Lukoyanov

**Affiliations:** 1 Institute of Physics and Technology, Ural Federal University Named after the First President of Russia B.N. Yeltsin, 620002 Ekaterinburg, Russia; 2 M.N. Mikheev Institute of Metal Physics of Ural Branch of Russian Academy of Sciences, 620108 Ekaterinburg, Russia

**Keywords:** electronic structure, topologic structure, alloys, intermetallics, first principles calculations

## Abstract

The electronic and band structures of ternary RNiSb and binary RSb compounds for R = Tb, Dy, Ho, have been investigated using an ab initio method accounting for strong electron correlations in the 4f shell of the rare-earth metals. These ternary compounds are found to be semiconductors with the indirect gap of 0.21, 0.21, and 0.26 eV for Tb, Dy, and Ho(NiSb), respectively. In contrast, in all binary RSb compounds, bands near the Fermi energy at the Г and X points are shifted relatively to RNiSb and form hole and electron pockets, so the energy gap is closed in RSb. The band structure typical for semimetals is formed in all RSb compounds for R = Tb, Dy, Ho. For the first time, we identify similar features near the Fermi level in the considered binary semimetals, namely, the presence of the hole and electron pockets in the vicinity of the Г and X points, the nonsymmetric electron pocket along Γ–X–W direction and hole pockets along the L–Γ–X direction, which were previously found experimentally in the other compound of this series GdSb. The magnetic moment of all considered compounds is fully determined by magnetic moments of the rare earth elements, the calculated effective magnetic moments of these ions have values close to the experimental values for all ternary compounds.

## 1. Introduction

The RTX is a family of compounds, where R is a rare earth element, T designates a transition metal, and X is a s/p element. These types of compounds have been known for their unusual electronic and magnetic properties, such as heavy fermion behavior [1,2], magnetic superconductivity [3,4], the presence of Weyl fermions [5], magnetocaloric effect [6,7,8], large magnetoresistance and negative temperature coefficient of resistivity [9,10], and more. Another outstanding characteristic of many RNiSb compounds is giant magnetoresistance [11,12] found in half-Heusler structure [13]. Such ternary materials with remarkable thermoelectric properties can be used for thermoelectric power generation and for conversion of waste heat in electricity with high efficiency [14]. High values of ZT, which is thermoelectric figure of merit, were observed in TmNiSb and Sn-doped ErNiSb with the value of 0.25 for TmNiSb at 700 K [15] and even higher value of 0.29 at 669 K for ErNiSb [16] and other compounds of this series [15,17,18,19]. 

Magnetic susceptibility measurements for RNiSb compounds have also been conducted and experiments show that most of the compounds exhibit Curie-Weiss behavior where magnetism is dominated by the magnetic moments of rare earth elements [20]. Theoretical electronic structure calculations for GdNiSb [21], as well as for the close GdNiGe compound [22], indeed show that the dominant contribution to the total magnetic moment is due to R while the contribution of Ni and Sb (Ge) is negligible. There is also a close binary compound GdSb that is found to have a pair of Weyl fermions, the presence of which can lead to chiral anomaly-induced negative longitudinal magnetoresistance under external magnetic field [23]. High-resolution angle-resolved photoemission spectroscopy (ARPES) measurements for DySb and HoSb were conducted and revealed at least two concentric hole pockets at the Γ point and two intersecting electron pockets at the X point [24], similar features were found in the band structure of LuSb calculated within GGA not accounting for electron correlations reported in [24]. Binary compound DySb was found to have extremely large positive magnetoresistance from experimental results with the suggested non-trivial band topology from DFT calculations with an inversion point seated about 0.34 eV below the Fermi level [25]. A recent experimental study of the binary compound HoSb also found extremely large magnetoresistance, the close interplay between conduction electrons and magnetism was suggested [26].

The ternary Tb/Ho/DyNiSb intermetallics, which are studied in this work, are half-Heusler compounds and crystallize in the cubic MgAgAs-type structure. This structure may be regarded as NaCl-type structure, where the rare earth and Sb atoms take the positions of the Na and Cl atoms, while the Ni atoms occupy one half of the tetrahedral voids formed by the Sb atoms [13]. It has also been shown that compounds in question exhibit large negative magnetoresistance at low temperatures which is caused by the reduction of spin disorder scattering due to the alignment of the moments under a magnetic field [11,12]. The ternary DyNiSb compound is found to be a narrow-gap intrinsic p-type semiconductor with the experimental value of the energy gap of 0.130–0.171 eV [9], 0.089–0.130 eV [15], for HoNiSb it is estimated as 0.08–0.11 eV [9]. 

In this study, we consider in detail the band (and electronic) structure, as well as magnetic properties, of the RSb and RNiSb compounds for R = Tb, Dy, Ho, in order to identify common topological features of the band structure, electronic structure and magnetic properties.

## 2. Materials and Methods

The ternary Tb/Dy/HoNiSb compounds have MgAgAs-type half-Heusler structure (space group F-43m, number 216) [13] with the lattice parameters: a = b = c = 6.304 Å for TbNiSb, a = b = c = 6.298 Å for DyNiSb and a = b = c = 6.262 Å for the HoNiSb compound [20] with following atomic positions Tb/Dy/Ho in 4*a* (0, 0, 0), Ni in 4*c* (1/4, 1/4, 1/4) and Sb in 4*b* (1/2, 1/2, 1/2). Binary RSb compounds have similar cubic crystal structure with the same atomic positions of rare earth element and Sb atoms with the following lattice parameters: a = b = c = 6.170 Å for TbSb, a = b = c = 6.150 Å for DySb and a = b = c = 6.130 Å for the HoSb compound [27].

**Figure 1:**
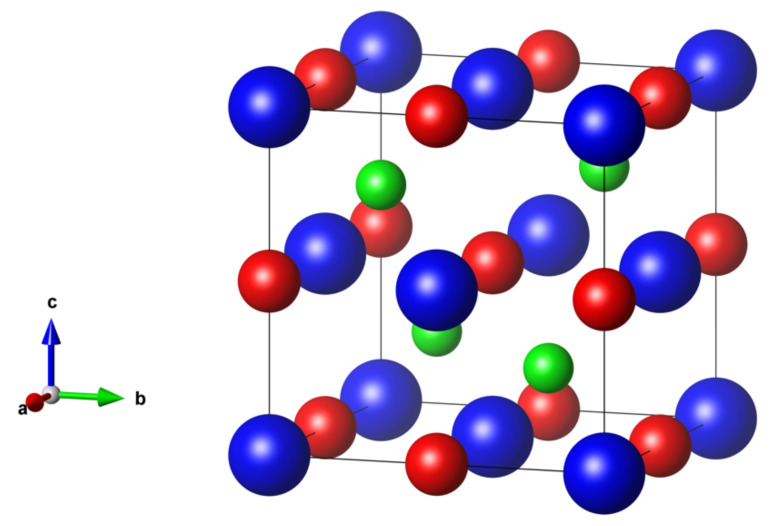
Crystal structure of RNiSb compounds. R atoms are shown in blue, Ni—in green, Sb—in red.

The crystal structure of Tb/Dy/HoNiSb is plotted in Vesta [28] in Figure 1. The unit cell of RNiSb contains 1 rare-earth atom, 1 nickel atom and 1 antimony atom. The Sb atom has an environment of four Ni atoms in the form of a tetrahedron. 

Electronic structure calculations were conducted in the Quantum Espresso package [29,30] using GGA+U version of LSDA+U method. Such method is widely used to take into account strong electron correlations between electrons of 4f shells in ions of rare earth elements. Parameters in GGA+U method have following values: Hund’s exchange parameter J = 0.7 eV for all three considered elements and direct Coulomb interaction U is equal to 5.4 eV for Tb, 5.8 eV for Dy and 5.9 eV for Ho [2,31]. In this work we assume that magnetic moments of rare earth elements have ferromagnetic ordering. The exchange correlation potential was employed in generalized gradient approximation (GGA) of Perdew-Burke-Ernzerhof (PBE) [32]. The calculations used the standard ultrasoft potentials from the pseudopotential library of Quantum ESPRESSO for Ni and Sb [33], projected augmented wave method (PAW) scalar-relativistic potentials for rare-earth elements from work [34]. Wave functions were expanded in plane waves, Bloechl’s tetrahedron method was employed for Brillouin-zone integration on a 12 × 12 × 12 k-point mesh, interactions between ions and valence electrons were taken into account within the framework of the method of plane augmented waves. 

## 3. Results

### 3.1. TbNiSb and TbSb Intermetallic Compounds 

**Figure 2:**
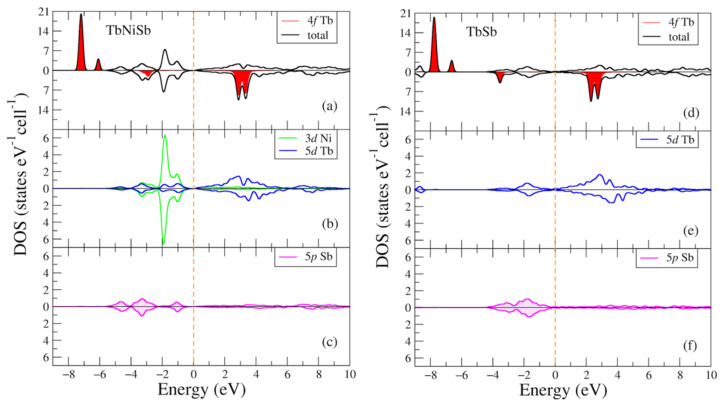
Densities of electronic states from DFT+U for (**a**–**c**) TbNiSb; (**d**–**f**) TbSb. (**a**,**d**) Total and partial Tb-4f densities of states; (**b**,**e**) Partial density of states for Tb-5d (Ni-3d); (**c**,**f**) Partial density of states for Sb-5p. The plot is shifted relatively to the Fermi energy shown at zero as a vertical line.

In Figure 2, the total and partial densities of electronic states of the TbNiSb and TbSb intermetallic compounds are given for two opposite spin directions. Two intense peaks in the total density of states for the majority spin direction of the TbNiSb (TbSb) compound in Figure 2a are formed by the 4f states of Tb at following energies: −7.2 (−7.8) eV and −6.1 (−6.6) eV below the valence band. Another two noticeable intense peaks in Figure 2a for the minority spin projection are found in the conduction band at energies 2.9 (2.3) eV and 3.3 (2.7) eV. One can see that valence band in TbNiSb compound is mostly formed by non-spin-polarized Ni 3d states Figure 2b with some contribution from Tb-5d and Sb-5p states. In binary TbSb compound the biggest contribution to the valence band is due to Sb-5p states Figure 2c. For both compounds and both spin projections Tb-5d states lay in the conduction band mostly unoccupied Figure 2b. The other electronic states are not shown in this figure due to their negligible contribution.

**Figure 3:**
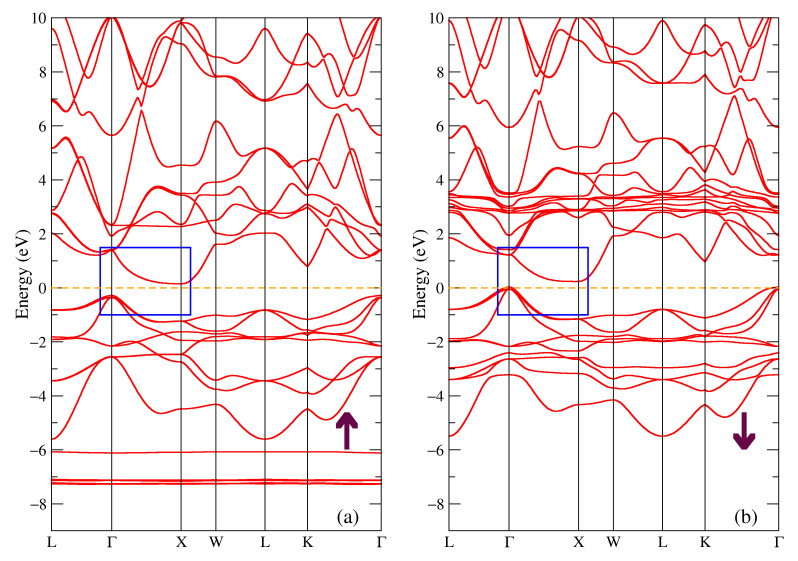
Band structure of TbNiSb: (**a**) majority and (**b**) minority spin projections. The blue rectangle points out the bands involved in the band gap formation.

The band structure for the majority and minority spin projections of TbNiSb is shown in Figure 3. One can see the energy gap of 0.43 eV in the majority spin projection in Figure 3a and energy gap of 0.21 eV for the other spin projection in Figure 3b. Point Г is the highest point in the valence band and the lowest point in the conduction band is X, so the compound is a semiconductor with an indirect gap. There are localized bands at the energies of eV −7.2 eV and −6.1 eV in Figure 3a and at the energies of (2.9; 3.3) eV in Figure 3b which correspond to intense peaks at the same energies in the density of states Figure 2a. There is also the presence of the occupied states near the Г point at the Fermi level.

**Figure 4:**
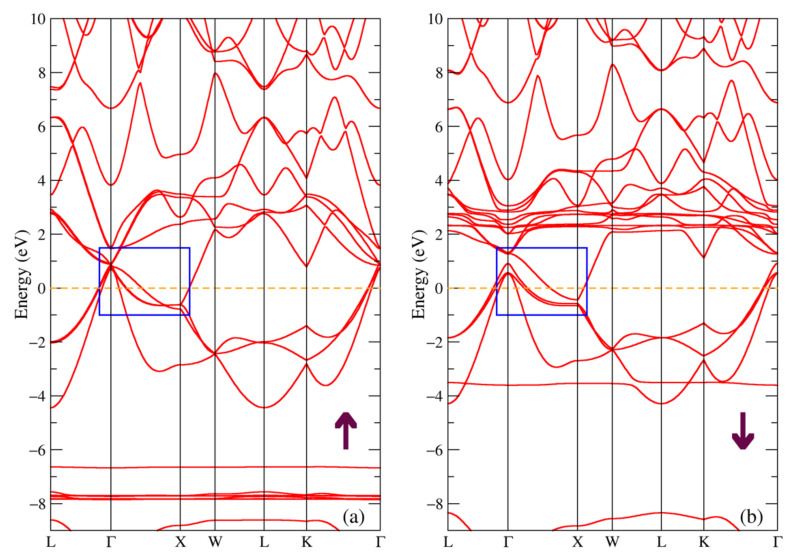
Band structure of TbSb: (**a**) majority and (**b**) minority spin projections. The blue rectangle points out the bands involved in the formation of pockets, see in the text.

The band structure for the majority and minority spin projections of TbSb is shown in Figure 4. The bands near the Fermi energy at the Г and X points are shifted relatively to the band structure of TbNiSb shown in Figure 3 and here they form hole and electron pockets (see the blue rectangle in Figure 4), so there is no energy gap for both spin projections. Such a band structure is typical for a semimetal. There is also a presence of similar to Figure 3 localized bands from 4f states of Tb at pretty much the same energies, only a few tenths of eV lower, one can find narrow intense peaks at these energies in the total density of states in Figure 2d.

### 3.2. DyNiSb and DySb Intermetallic Compounds 

**Figure 5:**
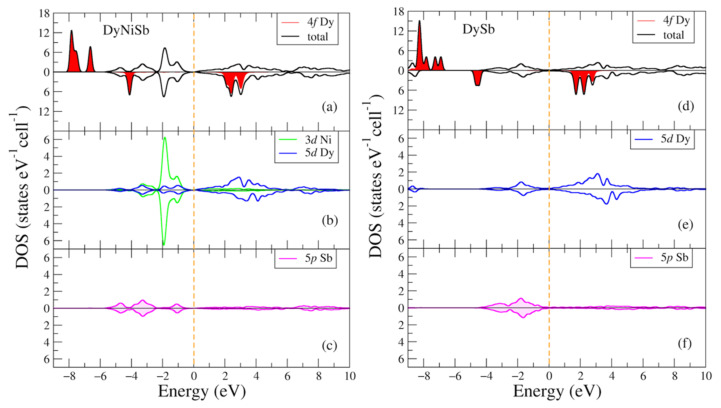
Densities of electronic states from DFT+U for (**a**–**c**) DyNiSb; (**d**–**f**) DySb. (**a**,**d**) Total and partial Dy-4f densities of states; (**b**,**e**) partial density of states for Dy-5d (Ni-3d); (**c**,**f**) partial density of states for Sb-5p. The plot is shifted relatively to the Fermi energy shown at zero as a vertical dashed line.

In Figure 5, the total and partial densities of electronic states of the DyNiSb and DySb intermetallic compounds are given for two opposite spin directions. In Figure 5a,d one can find similar to Figure 2a,d narrow intense peaks which manifest 4f states of rare earth metals. For the DyNiSb (DySb) compound and the majority spin projection such peaks are found at following energies: −7.8 (−8.3) eV and −6.6 (−7.3, −6.9) eV below the valence band. For the minority spin projection intense peaks are found at 2.4 (1.7) eV and 3.0 (2.2) eV. There is also a noticeable peak at −4.1 (4.6) eV contrary to Figure 2a,d for the minority spin projection for both DyNiSb and DySb in the bottom part of the valence band. The densities of Ni-3d, Dy-5d, and Sb-5p states in Figure 5 exhibit the behavior identical to those plotted in Figure 2.

**Figure 6:**
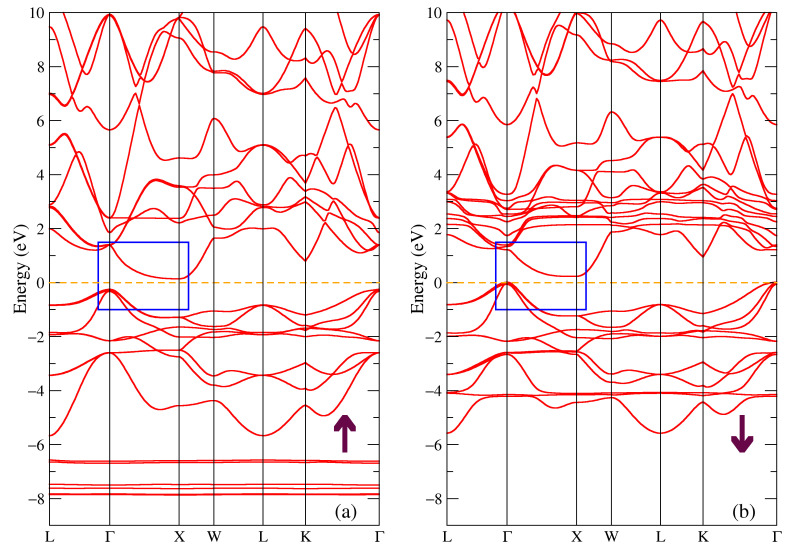
Band structure of DyNiSb: (**a**) majority and (**b**) minority spin projections. The blue rectangle points out the bands involved in the band gap formation.

The band structure for the majority and minority spin projections of DyNiSb compound is shown in Figure 6. The electronic states near the Fermi energy are mostly dominated by the Ni-3d and Dy-5d states, so the band structure near this level looks similar to the one in Figure 3 with the main difference being the value of the energy gap (see the blue rectangle in Figure 6). Thus, we conclude that the ternary DyNiSb compound is a semiconductor with an indirect gap of 0.39 eV for the majority spin projection and of 0.21 eV for the minority spin projection. It is in a good agreement with the experimental value of the energy gap of 0.130–0.171 eV [9], 0.089–0.130 eV [15] for DyNiSb. The localized bands from the 4f states of dysprosium similarly to previous Tb-compounds in Figure 3 and Figure 4 produce intense peaks at the same energies in the density of states in Figure 5a.

**Figure 7:**
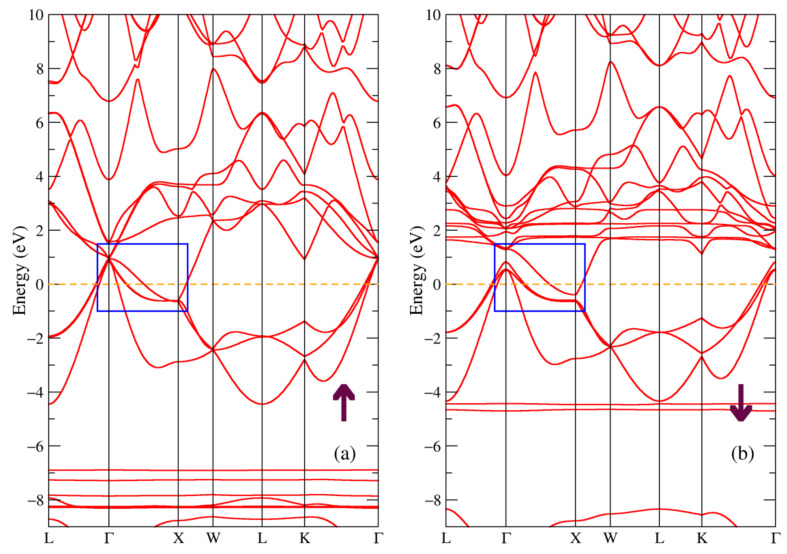
Band structure of DySb: (**a**) majority and (**b**) minority spin projections.

The band structure for DySb is shown in Figure 7. One can see that this picture is resembling Figure 4 where the band structure for the TbSb compound is shown, since the largest contribution to the states near the Fermi level is due to the Sb 5p and Dy 5d electronic states. The calculated band structure is similar to the one proposed for DySb in [25]. From Figure 7 and the blue rectangle pointing out the bands near the Fermi level, one can conclude that DySb is a semimetal with the hole and electron pockets in the band structure around Г and X points in the Brillouin zone.

### 3.3. HoNiSb and HoSb Intermetallic Compounds

**Figure 8:**
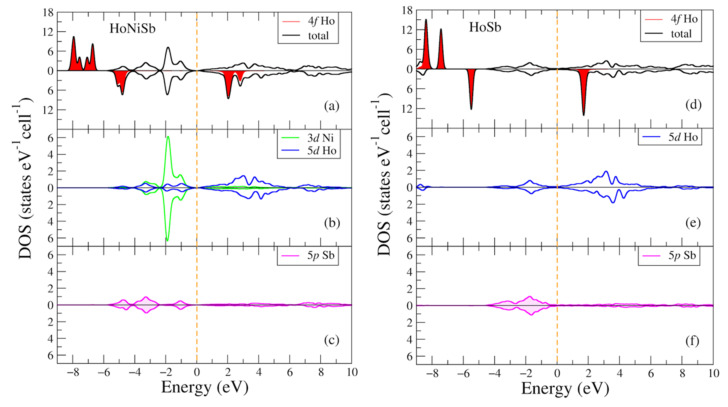
Densities of electronic states from DFT+U for (**a**–**c**) HoNiSb; (**d**–**f**) HoSb per cell. (**a**,**d**) Total and partial Ho-4f densities of states; (**b**,**e**) partial density of states for Ho-5d (Ni-3d); (**c**,**f**) partial density of states for Sb-5p. The plot is shifted relatively to the Fermi energy shown at zero as a vertical dashed line.

In Figure 8, the total and partial densities of electronic states of the HoNiSb and HoSb intermetallic compounds are given for two opposite spin directions. Densities of states for both compounds look similar to ones in Figure 2 and Figure 5 with the main difference being due to the holmium 4f shell. The positions of the intense peaks for this shell in the energy spectrum Figure 8a,d are following: −7.9 (−8.4) eV and −6.7 (−7.4) eV below the valence band for the majority spin projection and 2.0, 2.8 (1.7) eV for the minority spin projection. There is also another Ho-4f intense peak for the minority spin direction at −4.8 eV at the lower part of the valence band for the HoNiSb compound in Figure 8a, such as a peak which seems to be isolated from the Ho-5d and Sb-5p states in the binary HoSb compound at −5.5 eV (Figure 8d), it is not similar to that in the previous compounds.

**Figure 9:**
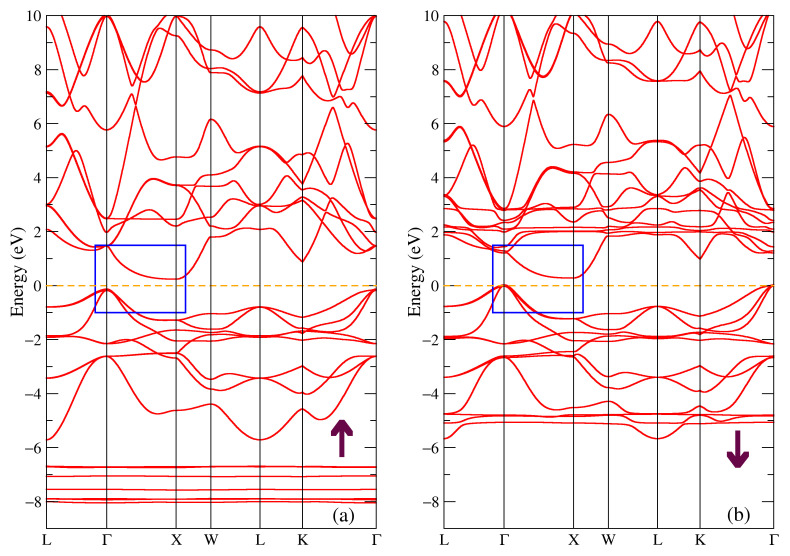
Band structure of HoNiSb: (**a**) majority and (**b**) minority spin projections. The blue rectangle points out the bands involved in the band gap formation.

The band structure for the majority and minority spin projections of HoNiSb compound is shown in Figure 9. Once again, we can see that ternary HoNiSb compound is a semiconductor with an indirect gap in the band structure resembling that of TbNiSb and DyNiSb in Figure 3 and Figure 6, respectively. The value for the energy gap is 0.37 eV for the majority spin projection and 0.26 eV for the minority spin projection. It is in a good agreement with the experimental value of the energy gap of 0.08–0.11 eV [9] for HoNiSb. Worth mentioning that the localized bands above the Fermi level from the 4f states of holmium are closest to the Fermi energy for HoNiSb in Figure 9b among all three ternary compounds. 

**Figure 10:**
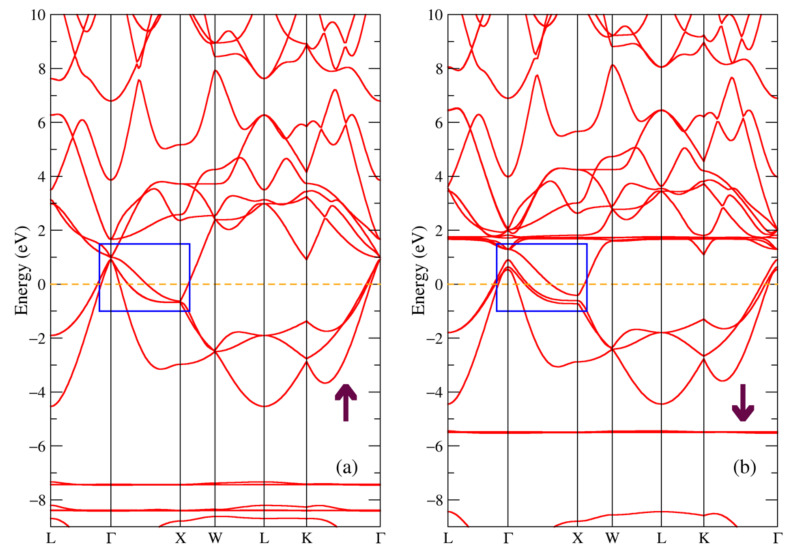
Band structure of HoSb: (**a**) majority and (**b**) minority spin projections.

The band structure for the majority and minority spin projections of the HoSb compound is shown in Figure 10. There is a noticeable flat band from the Ho-4f states in the minority spin projection Figure 10b only 1.8 eV above the Fermi level which is closest of all compounds. We can also see another localized band below the valence band in the minority spin projection which was discussed earlier. In the band structure near the Fermi level several bands are touching at X, see the blue rectangle in Figure 10. It is clearly seen that the binary HoSb compound is a semimetal with the band structure near the Fermi level very similar to that of the TbSb and DySb compounds shown in Figure 4 and Figure 7.

### 3.4. Magnetic Moments

In addition to the densities of states and band structures, the values of the magnetic moments of compounds were calculated within the framework of GGA+U. Magnetic moments of the nickel and stibium ions in the compounds are found to be negligible so we can consider it zero. Then all of the magnetic properties of considered RNiSb and RSb compounds are determined by those of rare earth elements [20]. The spin moments of rare earth ions, which are mostly defined by the 4f electronic states [22], as calculated in the present work, have following values: 5.92 $\mu_{B}$ for the Tb ions in both TbSb and TbNiSb compounds, 4.92 $\mu_{B}$ for Dy in DySb and DyNiSb compounds and 3.94 $\mu_{B}$ for the Ho ions in HoSb and HoNiSb. These values are close to the ones for the corresponding R^3+^ ions, but do not exactly coincide, see [20]. We can get the values of orbital momentum as for R^3+^ ions, L = 3, 5, and 6 for Tb, Dy, and Ho, respectively. Now, we are able to calculate the effective magnetic moments, and the results are 9.64 $\mu_{B}$ for TbSb and TbNiSb, 10.56 $\mu_{B}$ for DySb and DyNiSb, 10.55 $\mu_{B}$ for HoSb and HoNiSb vs. the experimental values reported as 10.2(4) $\mu_{B}$ for TbNiSb, 10.6(4) $\mu_{B}$ for DyNiSb, 10.7(4) $\mu_{B}$ for HoNiSb [20], 10.8 $\mu_{B}$ for HoSb [26].

## 4. Conclusions

In this work, we investigated the electronic and band structures of three ternary compounds Tb,Dy,Ho(NiSb) and three binary compounds Tb,Dy,Ho(Sb). Calculations were carried out in the framework of the GGA+U method and revealed that these ternary compounds are semiconductors with an indirect gap and the binary compounds are semimetals. The Tb,Dy,Ho(NiSb) semiconductors have the following values of the energy gap: 0.43, 0.39, and 0.37 eV for the majority spin projection and 0.21, 0.21, and 0.26 eV for the minority spin projection, respectively, which are in a good agreement with the published experimental values. From the band structure, we identify similar to each other topological features near the Fermi level in the Tb,Dy,Ho(Sb) binary semimetals, these are the hole and electron pockets in the vicinity of Г and X points, the non-symmetric electron pocket along Γ-X-W direction and hole pockets along the L-Γ-X direction. It is emphasized that the corresponding band structures can be found experimentally in the other compound of this series GdSb. It was also shown that the magnetic moment of all considered compounds is fully determined by magnetic moments of rare earth elements, the calculated effective magnetic moments of such ions have values close to experimental values.

## Data Availability

The data presented in this study are available on request from the corresponding author.
